# Experimental and Numerical Calculation of the Friction Performance of a Concrete Surface

**DOI:** 10.3390/ma16082989

**Published:** 2023-04-09

**Authors:** Shiren La, Chong Wang

**Affiliations:** 1 School of Civil and Traffic Engineering, Qinghai Nationalities University, Xining 810007, China; 2 School of Civil Engineering and Mechanics, Lanzhou University, Lanzhou 730000, China

**Keywords:** concrete pavement, surface roughness, temperature, spring slider model

## Abstract

The presented self-developed high-precision contact friction test device conducts experimental research on the friction characteristics of concrete pavement. First, the error analysis of the test device is carried out. The structure shows that the test device meets the test requirements. Subsequently, the device was used to carry out experimental research on the friction performance of concrete pavement under different roughness and temperature changes. The results showed that the friction performance of concrete pavement increased with the increase in surface roughness, and decreased with the increase in temperature. It has a small volume and significant stick-slip properties. Finally, the spring slider model is used to simulate the friction characteristics of the concrete pavement, then the shear modulus and viscous force of the concrete material are adjusted to achieve the calculation of the friction force over time under temperature changes, which is consistent with the experimental structure.

## 1. Introduction

Ice, sand and snow on roads in winter or cold regions have serious impact on the country’s traffic, economy and normal outdoor activities, and they are one of the main factors in the growth of the rate of road traffic accidents [1]. In addition, there is a direct relationship between vehicle driving stability and pavement skid resistance, and the skid resistance of asphalt pavement is affected by various uncertain factors over time. Because of its complex climate and working conditions, the plateau produces ice, sand and snow on the concrete pavement which make the friction phenomenon of the concrete pavement more complex [2]. In the early 20th century, researchers carried out studies on the mechanics and friction performance of concrete pavement, mainly focusing on the friction behavior of concrete surface, the friction damage model and anti-sliding characteristics [3,4,5]. Anti-sliding performance between tires and the road is an important index of driving safety, as well as the most important external representation of concrete roadbed surface friction effect [6,7]. However, skid resistance is mainly reflected in the characteristics of the contact between the tires and road surface, which is often accompanied by many complicated problems. It is mainly affected by the tires’ performance, grain, road roughness and contact with intermediate media. Clarifying the relevant mechanics and friction characteristics of the concrete pavement in this complex environment and in working conditions becomes a key factor in driving safety and stability, and accurately represents the calculation method of the dynamic friction coefficient in the complex medium of the plateau, i.e., the evaluation index of the anti-sliding performance. Contact mechanics models of tires and roads and the adhesion characteristics of pavement are of great importance for progress in relevant industries, and will provide important parameters for the effective use of concrete pavement and its process optimization.

For a better understanding of the frictional performance of concrete pavement and the main factors affecting its performance, we have systematically studied the contact characteristics between the tire and pavement. Grosch, Adam and other scholars have conducted a series of studies on the friction theory between tires and rough road surfaces, believing that the friction between road surfaces and rubber can be attributed to the comprehensive influence of adhesion and hysteretic deformation [8,9,10,11]. In Hertz [12]’s research on the contact mechanics of non-adhesive objects, he found that the external force is in a linear relationship with the contact stress, and it is related to the Poisson’s ratio and elastic modulus of the material. Persson and others [13] considered the influence of flash temperature in rubber tribology, random, anisotropic surfaces, and elastic lubrication layers in their research on contact mechanics, and verified the validity of the model by comparing it with experimental data. Ciavarella [14] simplified Persson’s multi-scale friction theory by considering viscoelastic loss of rubber, indicating that the wave vector is essentially a single-scale model with a fractal dimension D ≈ 2.2, and verifying the accuracy of the simplified model by referring to Lorenz theory. The results show that the energy dissipation caused by rubber sliding over the rough surface is the main cause of friction, and the adhesion between the rubber and fractal surface further leads to the generation of hysteresis force.

China has a 40-year history of research on concrete friction mechanisms and skid resistance evaluation, achieving remarkable results. For example, Guo Konghui et al., of Jilin University [15], proposed a test method of tire adhesion coefficients, and with the help of the newly developed tire adhesion test-bed, found the variation law of friction coefficient with slip rate, modified the previous tire steady-state semi empirical cornering model, and proposed a semi empirical model based on brush model and test data in 2007; this was called the unified tire model. The model can describe the relationship between tangential force and slip rate by exponential function and reflect the change in longitudinal force and lateral force with the friction state of road surface. In addition, Yang et al. [16] used a numerical simulation method to establish a high-speed vehicle driving model on wet pavement, and introduced the adhesion coefficient, including static and dynamic friction factors such as the anti-skid index, to analyze the anti-skid performance of asphalt pavement under the comprehensive action of vehicles and environment. A large number of studies show that the energy dissipation caused by rubber sliding across a rough surface remains the main cause of friction. It is believed that the adhesion between rubber and fractal surfaces further leads to the generation of hysteresis force.

Thanks to the endless efforts of scholars at home and abroad, research on the basic properties of concrete has achieved important success. However, the above studies have not systematically studied the mechanical properties and friction effects of the complex plateau environment (with changes in temperature and humidity) and working conditions (sand, freezing and wettability, etc.). In addition, the development of testing devices has a direct impact on the study of the key friction problems of concrete pavement, but there has been no substantial progress for a long time due to the lack of testing systems for mechanics and friction properties in complex plateau environments and working conditions.

## 2. Testing Device and Stability Analysis

### 2.1. Testing Device

**Figure 1:**
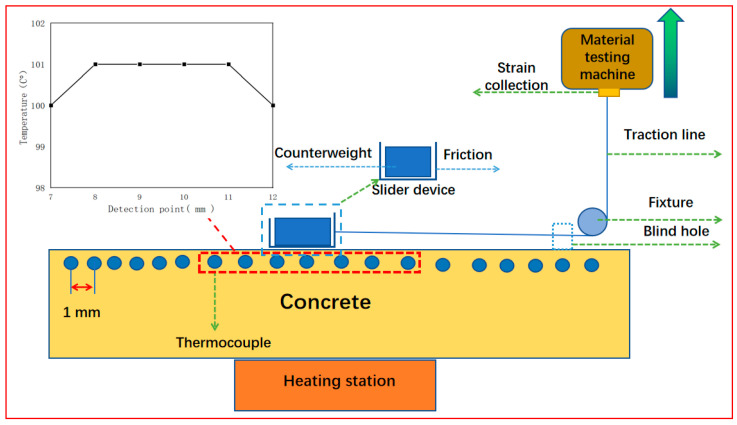
Temperature change concrete material friction performance test device.

In order to study the friction performance of concrete in a variable temperature environment, this study uses the test device shown in Figure 1. The device includes a heating table, fixed pulley, traction line, high-precision strain acquisition instrument, material testing machine, observation window, etc. Among them, a total of 20 temperature probes are arranged on the concrete surface to obtain the overall temperature of the concrete surface in the heating process. In addition, the device is equipped with observation windows and a high-resolution camera to observe the entire friction behavior of concrete blocks in real time. In this study, the heating table is placed in the center under the concrete, and the concrete surface temperature can be controlled by means of heat conduction. The temperature power of the heating table is 200 W, and the temperature is kept constant for 20 min when heated to 250 °C. The transverse temperature change in the concrete surface is as shown in Figure 1, in which the temperature of the second section of the concrete surface is lower, while the temperature of the middle section is higher and well distributed. The temperature of the middle 5 cm area is a constant value of 100 °C. Since the sliding distance of the slider is only 5 mm in the test process, the area polished by sandpaper can cover the whole sliding process of the slider. Therefore, the temperature of the concrete friction contact area is always constant after being treated by the heating table. In this paper, the author uses three temperatures to test. After heating through the heating table and ensuring that the temperature of the concrete middle area is constant, the test is carried out. In this paper, three temperatures are selected for testing: 50 °C, 70 °C and 100 °C.

**Figure 2:**
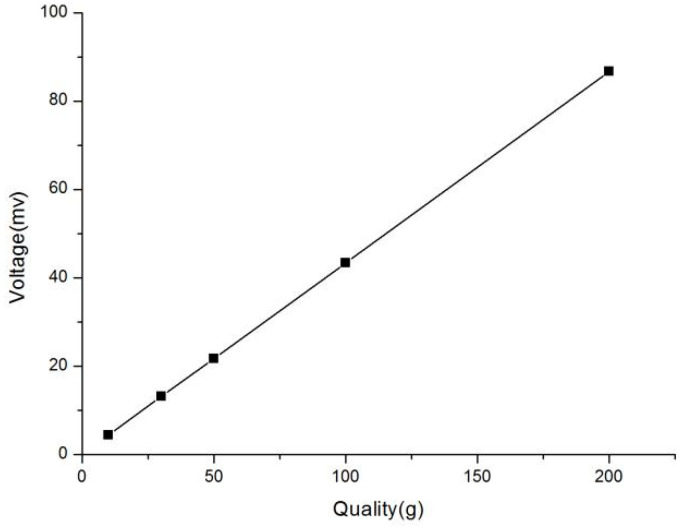
Variation curve of voltage value with weight of counterweight.

The device adopts fixed pulley transmission mode to produce the plane friction between the concrete roadbed surface and the sliding block. The sliding block and the high-precision strain sensor are connected by the highly elastic traction line so as to eliminate the influence of the deformation of the traction line on the test data as much as possible. The voltage value is obtained by hanging standard weights of different masses at the lower end of the strain sensor, and the corresponding relationship is found in order to accomplish the calibration of the strain collector. As shown in Figure 2, the voltage values corresponding to five different standard weights show a very good linear relationship, and the force resolution is 0.1 mN, which is accurate enough to meet the function of experimental measurement of material friction behavior. In the test, the positive pressure generated by the relative sliding process between the slider and the concrete block is controlled by changing the mass of the counterweight block, and the sliding speed of the material friction is controlled by changing the tensile rate of the material testing machine.

### 2.2. Stability Analysis

**Figure 3:**
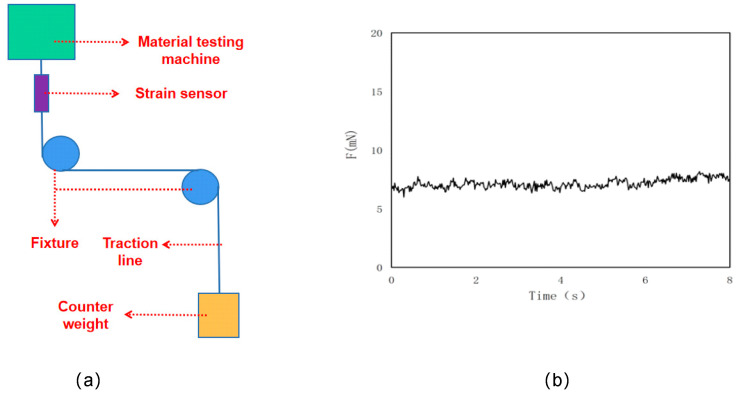
Schematic diagram of the process of producing the friction between the sliding block and the concrete surface through the friction between the traction wire and the fixed pulley. Among them, (**a**) is the calibration process of the test device, and (**b**) is the change curve of friction with time.

Given that the traction line produces the friction between the sliding block and the concrete surface through the fixed pulley, the main factor causing material friction is the direct friction between the traction line and the fixed pulley. This paper uses the test device shown in Figure 3a for calibration. The test conditions of the device are the same as those of the experimental device, and the counterweight is selected as the weight of the sliding block. Figure 3b shows the variation curve of the friction between the traction line and the fixed pulley with time. The results show that the friction directly generated by the traction line and the fixed pulley is stable. The value of the friction is 2.1 mN, and the fluctuation amplitude of the friction with time is only 0.3 mN. Therefore, in the study of the friction characteristics of the concrete surface, the direct friction between the traction line and the fixed pulley can be directly reduced, and this will not have a significant impact on the friction characteristics of the concrete.

## 3. Experimental Tests

### 3.1. Influence of Material Surface Roughness

**Figure 4:**
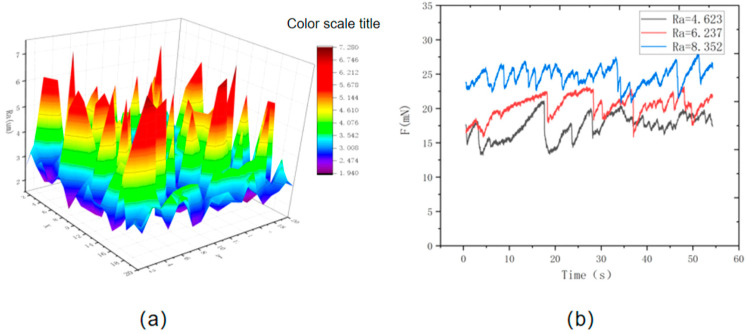
(**a**) The change in the surface roughness of the concrete after stabilization processing; (**b**) the change curve of the friction of the concrete material with different roughness.

Studies show that material’s surface roughness is the most critical factor affecting its friction characteristics [17,18,19,20]. Therefore, in this paper, the concrete surface is polished by sandpapers with different precision (three kinds of sandpaper with 2000 mesh, 5000 mesh and 10,000 mesh, respectively). Hard particles generated in the preparation process of coarse sandpaper are polished first, and then the surface is polished by fine sandpaper. The selected 20 mm×20 mm concrete surface area is divided into 400 small units, and then the surface roughness of the 400 units is measured by a contact roughness meter. Figure 4 shows the roughness of the treated concrete surface. In the figure, red is the area of high roughness whose maximum value is 5.268 μm, while green is the area of the lowest roughness whose minimum value is 3.721 μm, in which the average roughness of the material surface is 4.646 μm. The sliding block is made of rubber tire and rubber material with an elastic modulus of 7.8 MPa, and its surface is polished with sandpaper with an average roughness of 10 μm. There are many factors that affect the friction of materials. This paper focuses on the influence of temperature and surface roughness on the friction of a concrete surface by fixing the sliding rate and quality. Therefore, in this test, the relative sliding speed is 5 mm/min, and the mass of the slider is 500 g. It can be seen from the figure that the treated concrete surface is uneven, resulting in spikes due to the limited data collected. Since the sliding distance of the slider during the test is only within the range of a few millimeters, the area polished by sandpaper can cover the whole sliding process of the slider. In order to study the influence of the surface roughness of concrete materials on friction properties, average roughness values of 4.623 μm, 6.237 μm and 8.352 μm were obtained by grinding the surface of concrete materials with 2000 mesh, 5000 mesh and 10,000 mesh sandpaper. Figure 4 shows the variation curve of the friction of concrete materials with different roughness surfaces over time. When the roughness is 4.623 μm, the value of friction of concrete materials is 17.37 mN; when the roughness is 6.237 μm, the value of friction of concrete materials is 20.34 μm; when the roughness is 8.352 μm, the friction value of the concrete material is 24.67 mN. As a result, the friction value of the surface increases gradually with the increase in roughness, and the vibration amplitude of the friction also increases with the increase in roughness.

### 3.2. Influence of Temperature on Friction Properties of Concrete Materials

**Figure 5:**
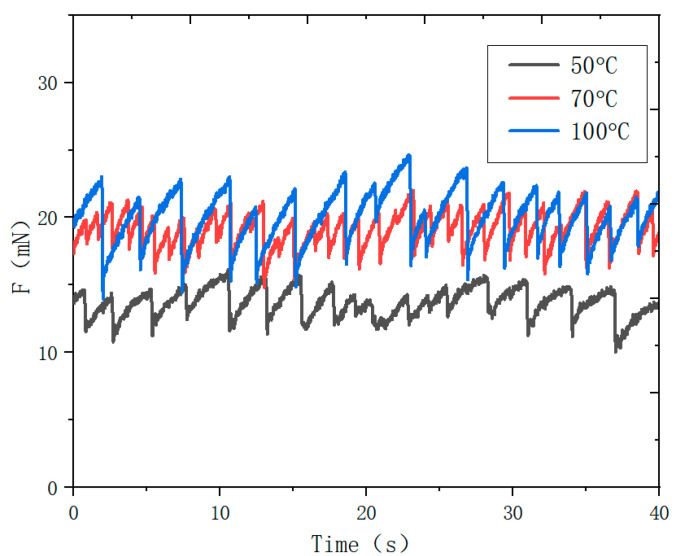
The variation curves of the friction of theconcrete material with time at three different temperatures.

Figure 5 shows the variation curve of concrete material friction with time under three temperatures. The results show that the value of friction increases gradually with the increase of temperature. The average friction is 13.61 mn when the concrete surface temperature is 50 °C, 19.14 mN when the temperature is 70 °C, and 20.05 mN when the temperature is 100 °C. In addition, it is obvious from the figure that the vibration amplitude of the friction increases gradually with time. The difference between the maximum and minimum frictions is 1.12 mN at 50 °C, 1.75 mN at 70 °C, and 2.29 mN at 100 °C. As the temperature of concrete material increases, the bonding property of concrete material improves. Therefore, the friction of the concrete material increases with the increase in temperature, and it has significant stick-slip characteristics.

## 4. Numerical Calculation

### 4.1. Model Establishment and Calculation

**Figure 6:**
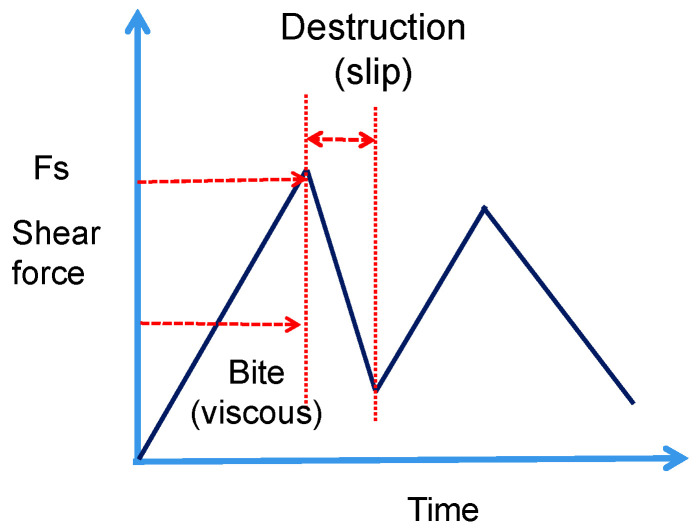
Curve of shear force variation with time in the process of the biting-destruction-batching of the microconvex body.

The material interface presents different forms at macro and micro levels. For example, the smooth and continuous material surface on the macroscopic level is composed of a large number of non-uniform and discrete concave convex bodies on the microscopic level, as shown in Figure 3. When the sliding block and the concrete surface are subjected to positive pressure, the micro-concave–convex will occlude (viscous), and when it is under traction action, the occluded (viscous) concave–convex may rupture. Additionally, this process depends on whether the local shear force has reached the maximum shear strength of the concave–convex. With the continuous movement of the slider, the broken (sliding) concave convex bodies are recombined and occluded (viscous) together, then the occlusion-destruction-occlusion-failure of a single microscopic concave–convex body shows an obvious stick-slip phenomenon, as shown in Figure 6. The cyclic behavior of the “occlusion-destruction-occlusion-destruction” of the microcosmic concave–convex body results in the propagation of shear force at the contact interface, which is also manifested as stick-slip phenomenon at macro scale after the evolution of complex microscopic behavior. Therefore, the sliding blocks are separated into numerous equal rigid sliding blocks, and each sliding block is connected by springs. The mass m of a single sliding block is the ratio of the mass of the sliding block to the number of sliding blocks. The equivalent stiffness of the sliding block is $k_{b}$, which is related to the elastic modulus of concrete and the segmented shape. Additionally, the adjacent slide blocks are connected by springs.

**Figure 7:**
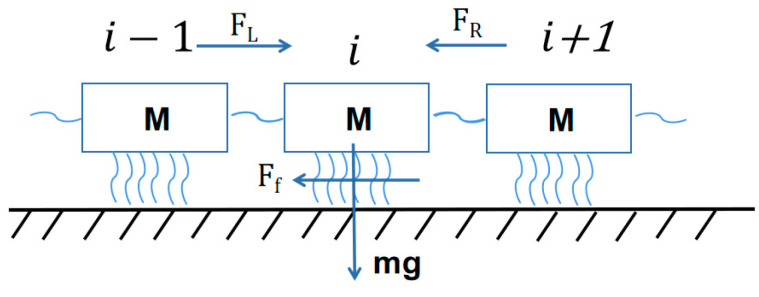
Concrete surface friction spring slider model.

In order to effectively simulate the action of the micro-concave–convex concrete interface, S interface springs are connected to each sliding block in the model, and the “connection-disconnection-connection” of springs is used to simulate the micro-behavior of micro-concave–convex concrete interface. The interface spring stiffness is $k_{i}$, $k_{i}=<k>{\left(f_{t}/\bar{f_{t}}\right)}^{2}$, where <k> is the average equivalent stiffness of interface link spring, which is related to the shear stiffness of interface material. $f_{t}$ is the strength of the interface spring, and the rough peak distribution of the interface contour is described by the Gaussian distribution. In view of the similarity between the rough peak and the concave–convex body, the strength distribution in the model can also be described by the Gaussian distribution. $\bar{f_{t}}$ is the ratio of the average shear strength of the interface spring, that is, the maximum shear strength of the sliding block (obtained experimentally) to the number of springs S. The whole sliding block system is only subjected to external force $T_{0}$ and T. $T_{0}$ is the force exerted by the counterweight block on the first sliding block, and T is the force exerted by the *n*th sliding block by the loading system. The dynamic analysis of the *i*th sliding block shows that it is subjected to the force of the left sliding block $F_{L}$ and the force of the right sliding block $F_{R}$. The damping coefficient of the slider is η. The damping force and the force of the sliding block are in the same form. The friction comes from the interface $F_{f}=\sum_{1}^{s}f_{j}$, where $f_{j}$ is the friction of the j spring and its own gravity mg. In order to effectively represent the effects of the cylinder interface micro-asperity, we use a connection-disconnection-connection model to simulate the micro-behavior of the contact interface asperity which was presented in [14,15]. The interface spring stiffness is $k_{i}=<k>{\left(f_{t}/\bar{f_{t}}\right)}^{2}$, where <k> denotes the average equivalent stiffness of the interface link spring and $f_{t}$ is the strength of the interface spring. Then, the dynamic equation of the whole spring slider system in the horizontal direction along the interface is expressed as follows:

(1) $$M\ddot{u}(t)+C\dot{u}(t)+Ku(t)=Q(t)$$

where M is the mass matrix, C is the damping matrix, K is the slider stiffness matrix, and Q is the external force of the system. The forces from the left and right sliders are perpendicular to the contact direction and the gravity of slider acts on the strength of interface spring $f_{t}$, thereby affecting the stiffness of interface connecting spring $k_{i}$. The number of divided sliders mainly affects the slider stiffness and shear strength. Here, we present a design schematic diagram to simulate the action of the micro-concave–convex body on the concrete interface, as shown in Figure 7.

**Figure 8:**
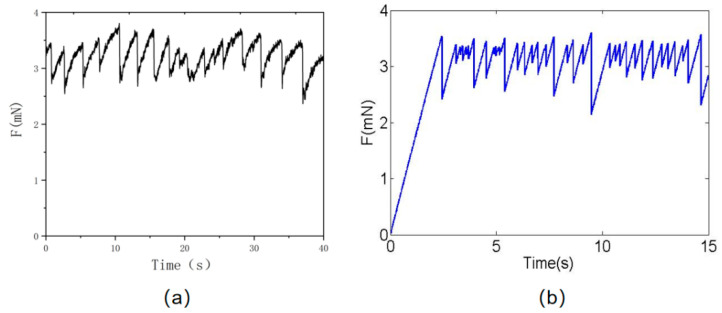
Friction performance of concrete surface: (**a**) experimental results; and (**b**) numerical simulation results.

Segmented sliding blocks mainly affect the slider stiffness and friction strength, and also change the number of sliding block models and the slider stiffness and friction strength at the same time. This has no effect on the final friction value. The more sliders there are, the longer the stick-slip time will be in the friction process; this is closer to the real experimental observation value, but the corresponding time is extremely long. In this paper, 50 sliding blocks are selected for calculation and the central difference method is used to solve the dynamic equation. The simulation results and experimental results are shown in Figure 8. The friction values of the experimental and simulated results are 3.21 Mn and 3.12 Mn, respectively, and the change curve of concrete surface friction with time shows an obvious stick-slip phenomenon. The numerical simulation results are consistent with the experimental test results.

### 4.2. Influence at Different Temperatures

**Figure 9:**
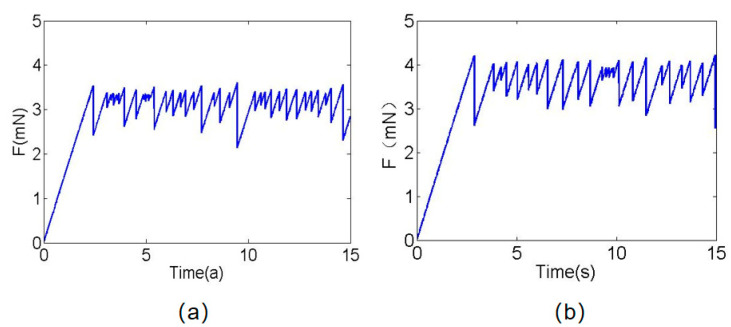
Friction curve change with time: (**a**) concrete surface friction curve at 50 °C, and (**b**) concrete surface friction curve at 100 °C.

The result shows that the shear modulus of concrete is mainly affected by temperature change. Its modulus decreases gradually with the increase in temperature, but its cohesiveness increases gradually with the increase in temperature. Therefore, in the spring slider model, changes in the friction characteristics of concrete surfaces under different temperatures are accomplished by adjusting the modulus of material and the initial traction force; in this way, the friction characteristics of concrete surfaces under temperature changes can be studied qualitatively. Figure 9a is the calculation result of the friction characteristics of concrete pavement at a temperature of 50 °C, and Figure 9b is the calculation result of the friction characteristics of concrete pavement at a temperature of 100 °C. It is found that the value of friction increases gradually with the increase in temperature, and it has a significant stick-slip characteristic, which is consistent with the experimental results.

## 5. Conclusions

Using the self-developed variable temperature environment, the testing device for the friction performance of a concrete surface was used to conduct experimental research on the friction behavior of concrete with different roughness and under different temperatures. It was found that the friction gradually increased with the increase in the roughness of material surface, and the vibration amplitude of friction also gradually increased. In addition, the value of friction increases gradually with the increase in temperature, but the vibration amplitude of friction does not change significantly with time. The frictional behaviors of concrete surface were simulated using a modified spring-slider model by taking into account the changes in the concrete’s surface roughness and temperature environment. The calculated results were consistent with the experimental results both qualitatively and quantitatively. The friction characteristics of the concrete surface at different temperatures are calculated by adjusting the modulus and initial traction of the material. The result shows that the value of friction and the vibration amplitude increase with time and with the increase in temperature, which is consistent with the experimental results.

## Data Availability

Not applicable.
